# Remarks on Proposed Amendments in the Lunacy Act Relating to Single Patients

**Published:** 1903-01-31

**Authors:** F. St. John Bullen


### REMARKS ON PROPOSED AMENDMENTS IN THE LUNACY ACT RELATING TO SINGLE PATIENTS.

By F. St. John Bullen, M.R.C.S.

(*Continued from p.* 281.)

The law as it stands undoubtedly leaves something to be desired with regard to the cases described by Sir W. Gowers, but it was devised for and has obtained an immense amount of safety for sane and insane persons. It has accomplished so much that there should be weighty evidence to show need of modifying its stringency, and it does not appear to us that the adoption of a similar notification system to that used in Scotland would meet the case, or, perhaps, even Dr. Gowers' views. His second proposition we cannot for a moment think would be accepted. We may ask, also, if there is so great a need for the alterations advocated. It would be desirable, we think, to feel sure that there is a sufficient number of cases mentally unsound who could receive benefit from treatment as borderland cases and to whom either unrestricted freedom or certification and detention would be prejudicial. And, again, it must be shown that the treatment of borderland cases at the present is unjustifiably hampered, as a matter of fact, by the action of the Commissioners. As to this last question, during the years 1895 to 1900 inclusive, prosecutions for illegally receiving persons of unsound mind were undertaken in 14 instances; in two of these the cases were dismissed (and it would seem improperly so), and in a third mere recognisances were demanded. Thus in six years in only 11 cases were prosecutions successful, and fines and costs ranging from £5 to £50 inflicted. And we think, with two or three exceptions, these actions were wisely taken by the Commissioners. No doubt it is only by reason of the information concerning such cases coming by chance and unofficial channels that there are so few prosecutions, for it is very probable that grounds exist under the present Act for a considerable number to be undertaken, and it would seem not unlikely that there is but little chance of the smallest trace of insanity escaping the notice and report of the Commissioners' visitor. At the same time it may be suggested that the infrequent prosecutions denote that the law is seldom infringed.

As there must always be some limitation imposed as to the cases classed as borderland (or notification cases) and those definitely insane and needing detention, it is necessary to form some idea as to those allowably fit for treatment under the first heading. Let it be granted that there are many persons who may be considered as of unsound mind and yet from the mildness of their mental disorder, preservation of control, absence of offensive characteristics, can be perfectly well managed without certification and its resultant restrictions. Such may be mild degrees of simple melancholia, hypochondriasis, amentia and dementia; some scarcely defined delusional states where the false ideas are inoffensive, unobtrusive, and without strong influence, rather of the nature of "crank" perhaps than decided delusion; also a corresponding type of hallucination, which can be certainly pronounced to have no tendency to produce any prejudicial effect on thought or action. Persons in fact, who can mix in ordinary family life and care for and protect themselves to a reasonable extent. On the contrary, cases which can only be treated properly under certificates include all those possibly dangerous to themselves or others; who might offend against order and decency; who could not manage or might mismanage their affairs, and any showing acute symptoms, lack of control, or insubordination under rational treatment and restrictions. Under this heading would come (*a*) all imbecile or demented persons unable to attend to themselves in such essential matters as feeding, clothing, cleanliness; who are unable to convey a knowledge of their ordinary requirements; who are uncontrolled in passion or propensity, or who require any notable degree of supervision to protect them from consequences of foolish or injurious acts; (*b*) any, even slight, degree of mania, especially if based on congenital defect is likely to give rise to trouble, but mania to the extent of marked inco-ordination in thought, speech, and action is unfit; (*c*) melancholia with definite loss of life-interest, serious apprehensiveness, refusal of food, loss of sleep and emaciation is unfit; (*d*) any delusion or hallucination which has control of conduct and which has any possible tendency to cause annoyance, to inflict direct or indirect harm to the patient or his surroundings, *e.g.*, delusions concerning property, ideas of definite persecution, of exaltation; hallucinations conveying ideas of bodily harm or annoyance, of insults, etc.; (*e*) insanity associated with sexual perversions, with chronic alcoholic excess, grave visceral disease, epilepsy, and (*f*) general paralysis. As has been indicated, where the Commissioners institute a prosecution, every available evidence of unsoundness of mind will be sought for, and it would probably be only a case well on the sane side of the borderline which would not be impeached. In the event, and we think a likely one, of the law regarding single patients not receiving alteration, we would advise persons intending to take borderland cases under their care to carefully weigh the nature of the symptoms of the intended inmate, to refuse any demanding coercive measures, however seldom, or, indeed, any treatment which does not allow of the patient's mixing to a reasonable extent with the household. Anyone accepting, without certificates, doubtful or decided cases of mental unsoundness, will be able to form an idea from the relative frequency of the prosecutions ordered by the Commissioners as to the risk they run, and also of the costs of an adverse decision.

